# Situational analysis on fluoroquinolones use and characterization of high-level ciprofloxacin-resistant *Enterococcus faecalis* by integrated broiler operations in South Korea

**DOI:** 10.3389/fvets.2023.1158721

**Published:** 2023-04-03

**Authors:** Yu Jin Lee, Hye-Ri Jung, Sunghyun Yoon, Suk-Kyung Lim, Young Ju Lee

**Affiliations:** ^1^College of Veterinary Medicine and Zoonoses Research Institute, Kyungpook National University, Daegu, Republic of Korea; ^2^Division of Microbiology, National Center for Toxicological Research, U.S. Food and Drug Administration, Jefferson, AR, United States; ^3^Bacterial Disease Division, Animal and Plant Quarantine Agency, Gimcheon-si, Gyeongsangbuk-do, Republic of Korea

**Keywords:** high level ciprofloxacin resistance, *Enterococcus faecalis*, broiler, Korea, biofilm, antimicrobial resistance genes, multidrug resistance, mutation

## Abstract

Fluoroquinolones are classified as “critically important antimicrobials for human medicine”; however, their extensive use in livestock poses a significant health risk to humans as it leads to the rapid spread of antimicrobial resistance. This study confirmed that 40.0%−71.4% of the farms in three of the five integrated broiler operations were administered ciprofloxacin (CIP). Moreover, preventive purposes (60.9%), veterinarian prescriptions (82.6%), drinking water route (100%), and 1 to 3 days (82.6%) of age were significantly highest (*P* < 0.05). 194 high-level ciprofloxacin-resistant (HLCR) *Enterococcus faecalis* (*E. faecalis*) were found in 65 of 74 farms, and of which, the prevalence of *qnrA* (63.9%), *tetM* (60.3%), *ermB* (64.9%), *blaz* (38.7%), and *catA* (34.0%) was significantly highest (*P* < 0.05). 154 (79.4%) isolates showed MDR, and the distribution of MDR was significantly differences among the operations (*P* < 0.05). All HLCR *E. faecalis* possessed double mutations in *gyrA* and *parC*, and S83I/S80I (90.7%) mutations were most commonly identified. Interestingly, the distribution of isolates with MICs ≥ 512 for both CIP and moxifloxacin was significantly higher in CIP–administered farms (56.5%) than in non-CIP–administered farms (41.4%) (*P* < 0.05). Also, the prevalence of strong or moderate biofilm formers in HLCR *E. faecalis* was significantly higher than that of weak and no biofilm formers (*P* < 0.05). HLCR *E. faecalis* were heavily distributed in the broiler farms in Korea; therefore, it is necessary to minimize the prevalence of resistant bacteria *via* structural management regulations such as cleaning and disinfection of farm environments.

## 1. Introduction

Antimicrobials play a critical role in the prevention and treatment of disease in humans and animals. However, the extensive use of antimicrobials poses a significant health risk to humans, leading to the rapid spread of antimicrobial resistance (1). Fluoroquinolones, including ciprofloxacin (CIP), are classified as “critically important antimicrobials for human medicine” by the World Health Organization (2), but they have been widely used for treatment and prevention of livestock because of their strong antibacterial activity against gram-negative bacteria, gram-positive bacteria, and mycoplasma (3). Fluoroquinolones inhibit DNA replication by inhibiting bacterial DNA topoisomerase (*parC* and *parE* subunits) and DNA gyrase (*gyrA* and *gyrB* subunits), which act as essential enzymes in bacteria (4). Therefore, single and double point mutations in the quinolone resistance determining region (QRDR) of these subunits reduce susceptibility and lead to high resistance to fluoroquinolones in the future (5, 6).

Because the risk promoting fluoroquinolones-resistant bacteria could be harmful to humans, US FDA has finally decided to withdraw approval of enrofloxacin for use in poultry in 2005 (7, 8), and in Ireland, fluoroquinolones have been prescribed in < 1% of antimicrobials for livestock since 2009 (9). Moreover, in Australia, fluoroquinolones have never been approved for food production animals due to strict regulations on antimicrobial use. Although the manufacture and import of enrofloxacin in the poultry industry in Korea have been only banned since October 2021 (10), the rate of resistance to CIP, a major metabolic product of enrofloxacin *in vivo*, increased by ~4.5% in 2021 compared with that in 2020 (10).

In Korea, 96.4% of the broilers are produced in integrated operations (11). However, antimicrobial resistance patterns may differ by operation depending on biosecurity and hygiene practices, vaccine programs, and antimicrobial applications (12). Therefore, this study was conducted to analyze the current status of fluoroquinolones-based prescriptions and characterize high-level CIP-resistant (HLCR) *E. faecalis* from five major integrated broiler operations which produce 50.2% of chicken meat in Korea (11).

## 2. Materials and methods

### 2.1. Data source

A situational analysis of CIP use was performed using a questionnaire on CIP administration patterns during the broiler grow-out period. The questionnaire areas included purpose, proponent, route, age, and duration of CIP administration.

### 2.2. Sample collection

Fecal and dust samples from 74 farms of five integrated broiler operations were collected in 2021 according to the standard set of Processing and Ingredients Specification of Livestock Products by the Ministry of Food and Drug Safety (2018) (13). Briefly, approximately 10 g of the dust was swabbed using a sterile gauze moistened with sterile double-strength skim milk, and feces were sampled to disposable boots in five different sites from each flock. All samples were placed in whirl pak bags (Whirl-Pak, Nasco, Madison, WI) and transported to the laboratory at 4°C.

### 2.3. Bacterial identification

The isolation and identification of *E. faecalis* were performed following the standard microbiological protocols published by the Ministry of Food and Drug Safety (2018). In brief, each dust and fecal samples were cultured in 90 mL of buffered peptone water (BPW; BD Biosciences, San Jose, CA, USA) for 18–24 h at 37°C. The pre-enriched BPW was transferred to Enterococcosel broth (BD Biosciences) at a ratio of 1:10 and streaked onto Enterococcosel agar (BD Biosciences) after incubation at 37 °C for 18–24 h. At least three presumptive *Enterococcus* spp. colonies were selected from each sample, and *E. faecalis* was confirmed using polymerase chain reaction (PCR) with a specific primer for *ddl*, as described previously (14). If two or more isolates from the same farm showed the same antimicrobial susceptibility pattern, only one isolate was selected. A total of 351 *E. faecalis* isolates were analyzed in this study.

### 2.4. Antimicrobial susceptibility testing

The minimum inhibitory concentrations (MICs) of CIP and moxifloxacin (MOX) were determined by standard agar dilution methods with Mueller–Hinton agar (BD Biosciences) according to the guidelines of the Clinical and Laboratory Standards Institute (15). The breakpoint for HLCR was set at ≥64 μg/mL against CIP (16). Moreover, based on the guidelines of the Clinical and Laboratory Standards Institute (15), antimicrobial resistance of all HLCR *E. faecalis* isolates was determined using the disc diffusion method with the following discs (BD Biosciences): ampicillin (10 μg), chloramphenicol (30 μg), ciprofloxacin (CIP, 5 μg), doxycycline (30 μg), erythromycin (15 μg), penicillin (10 units), rifampin (5 μg), tetracycline (30 μg), and vancomycin (30 μg). Multidrug resistance (MDR) was defined as the acquired non-susceptibility to at least one agent in three or more antimicrobial classes (17).

### 2.5. Identification of mutations in QRDRs

PCR was performed to amplify *gyrA* and *parC* in HLCR *E. faecalis* isolates using primers and conditions described previously (18). The amplified DNA was purified by a PCR purification kit (Bioneer Inc., Seoul, Republic of Korea) and sequenced using an automatic sequencer (Cosmogenetech, Seoul, Republic of Korea). The sequences were confirmed with those in the GenBank nucleotide database using the Basic Local Alignment Search Tool (BLAST) program available at the National Center for Biotechnology Information website (http://www.ncbi.nlm.nih.gov/BLAST).

### 2.6. Detection of antimicrobial resistance genes

DNA extraction was performed using the boiling method as described previously (19). The presence of resistance genes associated with fluoroquinolones (*qnrA, qnrB, qnrD*, and *qnrS*), β-lactams (*blaZ*, and *mecA*), phenicols (*cfr, catA*, and *catB*), glycopeptides (*vanA*), macrolides (*ermA, ermB*, and *ermC*), tetracyclines (*tetL, tetM*, and *tetO*), and rifamycin (*rpoB*) was detected using PCR as described previously (20–29).

### 2.7. Biofilm formation assay

All HLCR *E. faecalis* were determined for biofilm formation by using the standard microtiter plate test, as described previously (30). All HLCR isolates were cultured overnight on Brain–Heart Infusion agar (BD Biosciences, Sparks, MD, USA) at 37°C. Then, 500 μL of bacterial suspension adjusted for turbidity with 0.5 McFarland standard was inoculated into 5 mL of tryptic soy broth (BD Biosciences) with 0.25% (wt/vol) glucose, and 200 μL of the mixture was transferred into three wells of a 96-well microtiter plate. After overnight incubation at 37°C, the 96-well microtiter plates were washed with phosphate–buffered saline (BD Biosciences) to remove the planktonic bacteria. After air-drying, the attached bacteria were fixed with 200 μL of methanol for 15 min, and the bacterial biomass was quantified by measuring the absorbance at 595 nm (OD_595_) after staining with crystal violet (1% wt/vol) for 10 min and destaining with 50% ethanol-50% glacial acetic acid solution. The ability to form biofilms was classified as negative (OD_595_ < 0.12), weak (0.12 ≤ OD_595_ < 0.24), moderate (0.24 ≤ OD_595_ ≤ 0.48), and strong (OD_595_ > 0.48). To verify the analysis, *E. faecalis* OG1RF and *E. faecalis* ATCC 29212 were used as reference strains for weak and no biofilm formers, respectively. A sterile medium was used as the contamination control as described previously (31, 32).

### 2.8. Statistical analysis

Statistical analysis was performed using Pearson's chi-square tests and Fisher's exact tests with Bonferroni correction in Statistical Package for the Social Science version 25 (IBM, Korea). Significant differences were considered at *P* < 0.05.

## 3. Results

### 3.1. Analysis of the prescription status of CIP

The situational analysis of the prescription status of CIP in 74 farms of five integrated broiler operations is shown in Table 1. Among the five integrated broiler operations, the prevalence of CIP–administered farms was significantly highest in operation D (71.4%), followed by operations E (50.0%) and B (40.0%) (*P* < 0.05). No farms administered CIP in operations A and C. In the analysis for the purpose of CIP administration, operation D was significantly higher for the prevention (80.0%) than treatment (20.0%), whereas operation E were significantly higher for the treatment (60.0 %) than prevention (40.0 %) (*P* < 0.05). There were no significant differences between the rates of prevention and treatment in operation B. CIP administration *via* veterinarians (82.6%) and drinking water route (100%) was significantly highest (*P* < 0.05). Moreover, CIP was administered significantly highest at 1 to 3 days of age (82.6%), and significantly higher on duration of CIP administration in 2 days (43.5%) to 3 days (39.1%) (*P* < 0.05).

**Table 1:** Analysis of the prescription status of ciprofloxacin (CIP) by five integrated broiler operations.

**Variables**	**No. of farms included / No. of CIP–administered farms by five integrated broiler operations (%)**					
	**A**	**B**	**C**	**D**	**E**	**Total**
No. of CIP–administered farms/No. of farms tested (%)	0/15 (0.0)_c_	8/20 (40.0)_b_	0/15 (0.0)_c_	10/14 (71.4)_a_	5/10 (50.0)_b_	23/74 (31.1)
**Purpose of CIP administration**						
Prevention		4/8 (50.0)_b_		8/10 ${{\left(80.0\right)}_{\text{a}}}^{\text{A}}$	2/5 ${{\left(40.0\right)}_{\text{b}}}^{\text{B}}$	14/23 (60.9)^A^
Treatment		4/8 (50.0)_a_		2/10 ${{\left(20.0\right)}_{\text{b}}}^{\text{B}}$	3/5 ${{\left(60.0\right)}_{\text{a}}}^{\text{A}}$	9/23 (39.1)^B^
**Who proposed CIP**						
Veterinarian		7/8 ${{\left(87.5\right)}_{\text{a}}}^{\text{A}}$		9/10 ${{\left(90.0\right)}_{\text{a}}}^{\text{A}}$	3/5 ${{\left(60.0\right)}_{\text{b}}}^{\text{A}}$	19/23 (82.6)^A^
Veterinary drug dealer		1/8 (12.5)^B^		2/10 (20.0)^B^	2/5 (40.0)^B^	5/23 (21.7)^B^
Others*		0/8 (0.0)^C^		0/10 (0.0)^C^	0/5 (0.0)^C^	0 (0.0)^C^
**Route of CIP administration**						
Drinking water		8/8 (100.0)^A^		10/10 (100.0)^A^	5/5 (100.0)^A^	23/23 (100.0)^A^
Feed		1/8 ${{\left(12.5\right)}_{\text{a}}}^{\text{B}}$		0/10 ${{\left(0.0\right)}_{\text{b}}}^{\text{B}}$	0/5 ${{\left(0.0\right)}_{\text{b}}}^{\text{B}}$	1/23 (4.3)^B^
**Age of CIP administration (days)**						
1 ≤ ≤ 3		6/8 (75.0)^A^		9/10 (90.0)^A^	4/5 (80.0)^A^	19/23 (82.6)^A^
4 ≤ ≤ 6		2/8 (25.0)^B^		1/10 (10.0)^B^	1/5 (20.0)^B^	4/23 (17.4)^B^
15 ≤ ≤ 18		0/8 ${{\left(0.0\right)}_{\text{b}}}^{\text{C}}$		0/10 ${{\left(0.0\right)}_{\text{b}}}^{\text{C}}$	1/5 ${{\left(20.0\right)}_{\text{a}}}^{\text{B}}$	1/23 (4.3)^C^
**Duration of CIP administration (days)**						
1		1/8 ${{\left(12.5\right)}_{\text{a}}}^{\text{B}}$		2/10 ${{\left(20.0\right)}_{\text{a}}}^{\text{B}}$	0/5 ${{\left(0.0\right)}_{\text{b}}}^{\text{B}}$	3/23 (13.0)^B^
2		5/8 ${{\left(62.5\right)}_{\text{a}}}^{\text{A}}$		3/10 ${{\left(30.0\right)}_{\text{b}}}^{\text{B}}$	2/5 ${{\left(40.0\right)}_{\text{b}}}^{\text{A}}$	10/23 (43.5)^A^
3		2/8 ${{\left(25.0\right)}_{\text{b}}}^{\text{B}}$		5/10 ${{\left(50.0\right)}_{\text{a}}}^{\text{A}}$	2/5 ${{\left(40.0\right)}_{\text{a},\text{b}}}^{\text{A}}$	9/23 (39.1)^A^
4		0/8 ${{\left(0.0\right)}_{\text{b}}}^{\text{C}}$		0/10 ${{\left(0.0\right)}_{\text{b}}}^{\text{C}}$	2/5 ${{\left(40.0\right)}_{\text{a}}}^{\text{A}}$	2/23 (8.7)^B^

The superscript letter represents significant differences of the column, while the subscript letter represents significant differences of the row (*P* < 0.05).

^*^Others included farm owners and pharmaceutical company employees.

### 3.2. Distribution of HLCR *E. faecalis* and antimicrobial resistance genes

The distribution of HLCR *E. faecalis* and antimicrobial resistance genes in 74 farms of five integrated broiler operations are shown in Table 2. HLCR *E. faecalis* were found in 65 (87.8%) of 74 farms. In particular, HLCR *E. faecalis* were significantly higher at 95.0% and 100% in farms of operations B and E, respectively (*P* < 0.05). In addition, HLCR *E. faecalis* was found in a total of 194 (55.3%) of 351 *E. faecalis* isolates, which was significantly highest in farms of operation B (*P* < 0.05). In the distribution of fluoroquinolones resistance genes of HLCR *E. faecalis*, the prevalence of *qnrA* (63.9%) and *qnrB* (57.7%) genes were significantly higher than that of *qnrD* (20.1%) and *qnrS* (18.0%) (*P* < 0.05). In particular, *qnrA* showed a significantly high prevalence in the farms of four operations A (66.7%), B (60.7%), D (78.9%), and E (73.3%) except for operation C (45.0%) (*P* < 0.05), and *qnrB* was found in 50.0%−66.7% of farms in all five operations without significant differences.

**Table 2:** Distribution of high-level ciprofloxacin-resistant (HLCR) *E. faecalis* and antimicrobial resistance genes by five integrated broiler operations.

**Resistance gene**	**No. of isolates carried gene / No. of HLCR** ***E. faecalis*** **isolates by five integrated broiler operations (%)**					
	**A**	**B**	**C**	**D**	**E**	**Total**
No. (%) of farms with HLCR *E. faecalis* isolates / No. of farms tested (%)	11/15 (73.3)_b_	19/20 (95.0)_a_	12/15 (80.0)_b_	13/14 (92.9)_a, b_	10/10 (100.0)_a_	65/74 (87.8)
No. of HLCR *E. faecalis* isolates / No. of *E. faecalis* isolates (%)	30/68 (44.1)_b_	56/81 (69.1)_a_	40/79 (50.6)_b_	38/65 (58.5)_a, b_	30/58 (51.7)_a, b_	194/351 (55.3)
*qnrA*	20/30 (66.7)_a_	34/56 (60.7)_a, b_	18/40 (45.0)_b_	30/38 (78.9)_a_	22/30 (73.3)_a_	124/194 (63.9)^A^
*qnrB*	17/30 (56.7)	30/56 (53.6)	20/40 (50.0)	25/38 (65.8)	20/30 (66.7)	112/194 (57.7)^A^
*qnrD*	3/30 (10.0)_b_	11/56 (19.6)_a, b_	8/40 (20.0)_a, b_	9/38 (23.7)_a, b_	8/30 (26.7)_a_	39/194 (20.1)^B^
*qnrS*	5/30 (16.7)	8/56 (14.3)	8/40 (20.0)	6/38 (15.8)	8/30 (26.7)	35/194 (18.0)^B^
No. (%) of tetracyclines resistant HLCR *E. faecalis* isolates	25/30 (83.3)	48/56 (85.7)	34/40 (85.0)	29/38 (76.3)	23/30 (76.7)	159/194 (82.0)
*tetL*	12/30 (40.0)_a, b_	25/56 (44.6)_a_	10/40 (25.0)_b_	12/38 (31.6)_a, b_	10/30 (33.3)_a, b_	69/194 (35.6)^B^
*tetM*	20/30 (66.7)	32/56 (57.1)	22/40 (55.0)	25/38 (65.8)	18/30 (60.0)	117/194 (60.3)^A^
*tetO*	1/30 (3.3)	0/56 (0.0)	0/40 (0.0)	0/38 (0.0)	0/30 (0.0)	1/194 (0.5)^C^
No. (%) of macrolides resistant HLCR *E. faecalis* isolates	22/30 (73.3)	48/56 (85.7)	30/40 (75.0)	30/38 (78.9)	24/30 (80.0)	154/194 (79.4)
*ermA*	1/30 (3.3)	1/56 (1.8)	2/40 (5.0)	3/38 (7.9)	3/30 (10.0)	10/194 (5.2)^B^
*ermB*	19/30 (63.3)	40/56 (71.4)	24/40 (60.0)	25/38 (65.8)	18/30 (60.0)	126/194 (64.9)^A^
*ermC*	0/30 (0.0)	0/56 (0.0)	0/40 (0.0)	0/38 (0.0)	0/30 (0.0)	0/194 (0.0)^B^
No. (%) of β-lactams resistant HLCR *E. faecalis* isolates	13/30 (43.3)	22/56 (55.4)	16/40 (50.0)	15/38 (50.0)	14/30 (60.0)	101/194 (52.1)
*blaZ*	10/30 (33.3)	23/56 (41.1)	15/40 (37.5)	14/38 (36.8)	13/30 (43.3)	75/194 (38.7)^A^
*mecA*	2/30 (6.7)	1/56 (1.8)	2/40 (5.0)	0/38 (0.0)	1/30 (3.3)	6/194 (3.1)^B^
No. (%) of phenicols resistant HLCR *E. faecalis* isolates	10/30 (33.3)	24/56 (42.9)	18/40 (45.0)	16/38 (42.1)	16/30 (53.3)	84/194 (43.3)
*cfr*	2/30 (6.7)	2/56 (3.6)	0/40 (0.0)	2/38 (5.3)	0/30 (0.0)	6/194 (3.1)^B^
*catA*	12/30 (40.0)	18/56 (35.7)	13/40 (45.0)	12/38 (47.4)	11/30 (36.7)	66/194 (34.0)^A^
*catB*	2/30 (6.7)	1/56 (1.8)	0/40 (0.0)	2/38 (5.3)	1/30 (3.3)	6/194 (3.1)^B^
No. (%) of rifamycin resistant HLCR *E. faecalis* isolates	10/30 (33.3)	16/56 (28.6)	15/40 (37.5)	12/38 (31.6)	10/30 (33.3)	63/194 (32.5)
*rpoB*	10/30 (33.3)	13/56 (23.2)	13/40 (32.5)	9/38 (23.7)	8/30 (26.7)	53/194 (27.3)
No. (%) of glycopeptides resistant HLCR *E. faecalis* isolates	1/30 (3.3)	0/56 (0.0)	0/40 (0.0)	0/38 (0.0)	1/30 (3.3)	2/194 (1.0)
*vanA*	0/30 (0.0)	0/56 (0.0)	0/40 (0.0)	0/38 (0.0)	0/30 (0.0)	0/194 (0.0)
No. (%) of multidrug resistant HLCR *E. faecalis* isolates	22/30 (73.3)_b_	49/56 (87.5)_a_	28/40 (70.0)_b_	30/38 (78.9)_a, b_	25/30 (83.3)_a, b_	154/194 (79.4)

The superscript letter represents significant differences of the column, while the subscript letter represents significant differences of the row (*P* < 0.05).

All HLCR *E. faecalis* also showed the highest resistance to tetracyclines (82.0%), followed by macrolides (79.4%), β-lactams (52.1%), phenicols (43.3%), rifamycin (32.5%), and glycopeptides (1.0%) without no significant differences among the five integrated broiler operations. Moreover, the prevalence of *tetM* (60.3%), *ermB* (64.9%), *blaz* (38.7%), and *catA* (34.0%) was significantly highest among tetracyclines, marcrolides, β-lactams and phenicols resistance-related genes, respectively (*P* < 0.05). MDR was found in 154 (79.4%) of 194 HLCR *E. faecalis* isolates; however, the distribution of MDR was significantly highest in operation B (87.5%) and lowest in operations A (73.3%) and C (70.0%) (*P* < 0.05).

### 3.3. Detection of mutations in the QRDRs of HLCR *E. faecalis*

The presence of mutations in *gyrA* and *parC* and distribution of MICs in 194 HLCR *E. faecalis* isolates are shown in Table 3. All HLCR *E. faecalis* isolates possessed double mutations in *gyrA* and *parC*. Moreover, S83I/S80I (90.7%) mutations were most commonly identified. The distribution of isolates with MICs ≥ 512 for both CIP and MOX was significantly higher in CIP–administered farms (56.5%) than in non-CIP–administered farms (41.4%) (*P* < 0.05).

**Table 3:** Characteristics of amino acid changes in *gyrA* and *parC* and distribution of MICs in 194 high-level ciprofloxacin-resistant (HLCR) *E. faecalis* from 74 broiler farms of five integrated broiler operations.

**Amino acid change**		**MIC (**μ**g/mL)**^**a**^		**No. (%) of HLCR** ***E. faecalis*** **isolates**		
***gyrA***	***parC***	**CIP**	**MOX**	**CIP–administered farms (*****n*** = **124)**	**Non–CIP–administered farms (*****n*** = **70)**	**Total (*****n*** = **194)**^b^
S83I	S80I	≥512	≥512	70 (56.5)_a_	29 (41.4)_b_	99 (51.0)
		256	≥512	17 (13.7)	14 (20.0)	31 (16.0)
		≥512	256	11 (8.9)	11 (15.7)	22 (11.3)
		256	256	6 (4.8)	6 (8.6)	12 (6.2)
		256	128	2 (1.6)	3 (4.3)	5 (2.6)
		128	256	2 (1.6)	0 (0.0)	2 (1.0)
		128	128	2 (1.6)	0 (0.0)	2 (1.0)
		128	64	1 (0.8)	0 (0.0)	1 (0.5)
		64	64	1 (0.8)	1 (1.4)	2 (1.0)
S83F	S80I	≥512	256	5 (4.0)	2 (2.9)	7 (3.6)
		256	256	2 (1.6)	1 (1.4)	3 (1.5)
		256	128	2 (1.6)	0 (0.0)	2 (1.0)
		128	64	2 (1.6)	1 (1.4)	3 (1.5)
S83V	S80I	≥512	256	1 (0.8)	0 (0.0)	1 (0.5)
S83Y	S80I	≥512	256	0 (0.0)	1 (1.4)	1 (0.5)
S83I	S84K	256	256	0 (0.0)	1 (1.4)	1 (0.5)

^a^MIC, Minimum Inhibitory concentrations; CIP, ciprofloxacin; MOX, moxifloxacin.

^b^All isolates possessed double mutations in *gyrA* and *parC*.

The subscript letter represents significant differences of the row (*p* < 0.05).

### 3.4. Distribution of biofilm-forming ability of HLCR *E. faecalis*

The distribution of biofilm-forming ability of 194 HLCR *E. faecalis* isolates is shown in Table 4. Although there were no significant differences between CIP–administered farms and non-CIP–administered farms, the prevalence of strong or moderate biofilm formers (60.5% and 60.0%, respectively) in all HLCR *E. faecalis* showed significantly higher than that of weak and no biofilm formers (*P* < 0.05).

**Table 4:** Distribution of biofilm-forming ability in 194 high-level ciprofloxacin-resistant (HLCR) *E. faecalis* from 74 broiler farms of five integrated broiler operations.

**Biofilm former**	**No. (%) of HLCR** ***E. faecalis*** **isolates**		
	**CIP–administered farms (*****n*** = **124)**	**Non-CIP–administered farms (*****n*** = **70)**	**Total (*****n*** = **194)**
No biofilm former	19 (15.3)^B^	10 (14.3)^B^	29 (14.9)^B^
Weak biofilm former	30 (24.2)^B^	18 (25.7)^B^	48 (24.7)^B^
Strong or moderate biofilm former	75 (60.5)^A^	42 (60.0)^A^	117 (60.3)^A^

The superscript letter represents significant difference of the column (*p* < 0.05).

## 4. Discussion

Enterococci are harmless to healthy individuals, but are considered opportunistic pathogens associated with hospital-acquired infections in humans and animals (33). In particular, the ability to cause infection is critical to avoid the action of most commonly used antimicrobials. Recently, antimicrobial-resistant enterococci isolated from poultry farms have become a problem in many countries, including Korea (34–37), because antimicrobial-resistant enterococci can easily spread within pathogenic strains or between pathogenic and non-pathogenic strains (38). Moreover, resistance to fluoroquinolones, which are critically important in human medicine, has been continuously reported worldwide (39).

Antimicrobial resistance in enterococci is described as intrinsic resistance found within a species genome as well as acquired resistance by obtaining new genetic material or acquiring sporadic mutations in intrinsic genes (40, 41). In particular, the prevalence of acquired fluoroquinolones-resistant enterococci has increased with the increased use of fluoroquinolones (36, 42). In this study, CIP was administered in 23 (31.1%) of 74 broiler farms of five integrated broiler operations. Two integrated broiler operations did not use CIP, but the other three used CIP for prevention and treatment, mostly under the proposal of veterinarian. Moreover, the prevalence of 1 to 3 days of age (82.6%) was the highest among the age of CIP administration in the three integrated broiler operations. It is related to the high incidence of colibacillosis in young chicks causing mortality in Korea (43). CIP was also mainly administered *via* drinking water for 2 or 3 days because it is easier than injection administration, but it has a disadvantage that the time when the drug appears varies depending on individual even within the same flock (44).

In this study, HLCR *E. faecalis* were isolated in 65 (87.8%) of 74 farms. Farms of operations A and C were not administered CIP at all, but the distribution of HLCR *E. faecalis* isolates in these two farms showed a high prevalence of 73.3% and 80.0%, respectively. Burbarelli et al. (45) reported that antimicrobial–resistant bacteria could survive and spread throughout the breeding period regardless of CIP administration unless cleaning and disinfection of the broiler environment is followed for farm hygiene. In particular, antimicrobial–resistant bacteria in feces, which are generally considered a natural reservoir, have the potential to remain in the environment or bind to dust and spread throughout the farm (46–48).

Moreover, in this study, the antimicrobial resistance genes *qnrA* and *qnrB*, which are related to fluoroquinolones resistance, were present in 63.9% and 57.7%, respectively, of the distribution of HLCR *E. faecalis* in the five integrated broiler operations. In particular, the prevalence of *qnrA* in HLCR *E. faecalis* isolates in operation A was 66.7%, but the prevalence of *qnrA* in operation C was 45.0%, which was significantly lowest in these operations, even though CIP was not administered in operation A and C. It is thought that environmental cleaning and the use of CIP, and it is necessary to continuously investigate the distribution of these genes in farms that do not use CIP.

The plasmids carrying mobile resistance genes are easily transferable from one bacteria to another by conjugation, sharing genetic information (49, 50). Moreover, the externalization of antimicrobials *via* efflux pumps responsible for the outflow of the antimicrobials can simultaneously confer resistance to several antimicrobials, and their overexpression can lead to high levels of resistance and MDR (51–53). In this study, HLCR *E. faecalis* isolated from five broiler integrated operations were mostly resistant to tetracyclines (82.0%), macrolides (79.4%), β-lactams (52.1%), phenicols (43.3%), and rifamycin (32.5%), without significant differences among the operations. In addition, the prevalence of *tetM, ermB, blaZ, catA*, and *rpoB*, which are related to resistance to tetracyclines, macrolides, β-lactams, and phenicols, was 60.3%, 64.9%, 38.7%, and 34.0%, respectively, without significant differences among the operations. However, the distribution of MDR was significantly highest in operation B (87.5%) and lowest in operations A (73.3%) and C (70.0%). These results are consistent with the fact that the distribution of HLCR *E. faecalis* isolates was significantly highest in farms of operation B and lowest in farms of operations A and C. Moreover, this is consistent with the reports that the efflux pump is overexpressed as the number of HLCR isolates increases (54, 55), which is thought to be related to the increased distribution of MDR.

The mechanism of resistance to fluoroquinolones includes the formation of mutations in the two bacterial essential enzymes, DNA gyrase and DNA topoisomerase (56). DNA gyrase comprises *gyrA* and *gyrB* subunits, and DNA topoisomerase comprises *parC* and *parE* subunits. In particular, HLCR is associated with double mutations of *gyrA* and *parC* (57). Kim et al. (36) reported that all tested HLCR enterococci isolates showed double mutations in *gyrA* and *parC*, and the double mutations were the most frequently identified in S83I/S80I at 94.1%. Similarly, all HLCR *E. faecalis* isolates in this study possessed double mutations in *gyrA* and *parC*. In particular, 90.7% of HLCR isolates showed S83I/S80I mutations. Moreover, 99 (56.3%) of 176 HLCR *E. faecalis* with S83I/S80I mutations showed MICs of ≥512 for CIP and MOX. However, the distribution of these HLCR *E. faecalis* with MIC of ≥512 for both CIP and MOX in S83I/S80I mutations was found to be significantly higher in CIP–administered farms (56.5%) than in non–CIP–administered farms (41.4%). These results indicate that higher CIP administration leads to higher MIC values, just as fluoroquinolones-resistant enterococci increase with increasing fluoroquinolones use.

Biofilms of bacteria protect them from host immunological responses, phagocytosis, and antimicrobials (58). The ability to form biofilms is related to the ability of bacteria to adhere to a surface and form a layer, and the density of the layer indicates the strength of the biofilms (59). In addition, the thicker the layer, the less effective the antimicrobials against bacteria, suggesting a correlation between antimicrobial resistance and biofilm-forming ability (60). Al-Shammary (61) reported that 93.8% of MDR *E. faecalis* isolates from raw milk samples were biofilm formers. Sadek and Koriem (62) also reported that all MDR *E. faecalis* isolates from dairy products were biofilm formers. In this study, 165 (85.0%) of 194 HLCR *E. faecalis* isolates showed strong or moderate biofilm-forming ability. However, there were no significant differences between the isolates from CIP–administered farms and non–CIP–administered farms. Therefore, these results suggest that if already classified as HLCR, isolates can have strong and moderate biofilm-forming ability regardless of CIP administration, and the increased biofilm density also allows infections to persist chronically despite antimicrobial treatment strategies for broiler diseases. In this study, it was confirmed that HLCR *E. faecalis* were heavily distributed in the broiler operations in Korea, therefore, it is necessary to minimize the prevalence of resistant bacteria *via* structural management regulation, such as cleaning and disinfection of farm environments, and reduce the use of antimicrobials *via* prescriptions based on scientific disease evidence rather than disease prevention.

## Data availability statement

The original contributions presented in the study are included in the article/supplementary material, further inquiries can be directed to the corresponding author.

## Author contributions

YuL, SK-L, and YoL conceived of the presented idea. YuL, H-RJ, and SY developed the theory and performed the experiments. YuL and H-RJ verified the analysis. YuL wrote the manuscript with supervision from S-KL and YoL. All authors contributed to the article and approved the submitted version.
